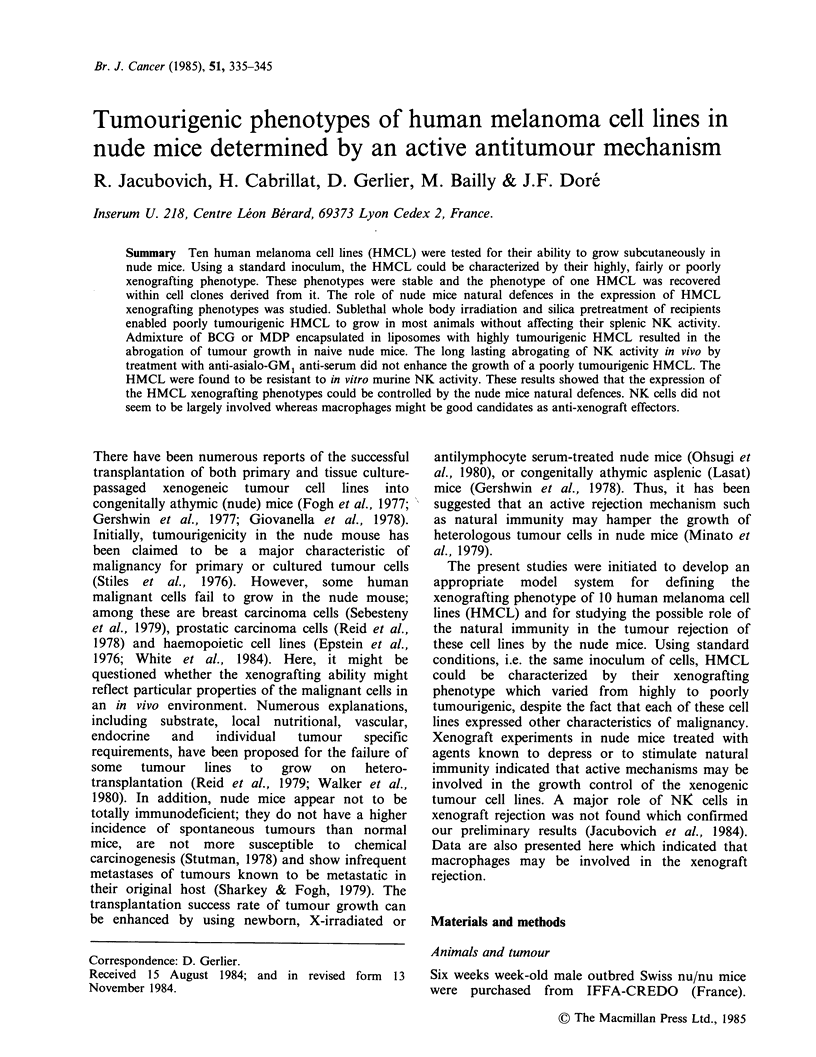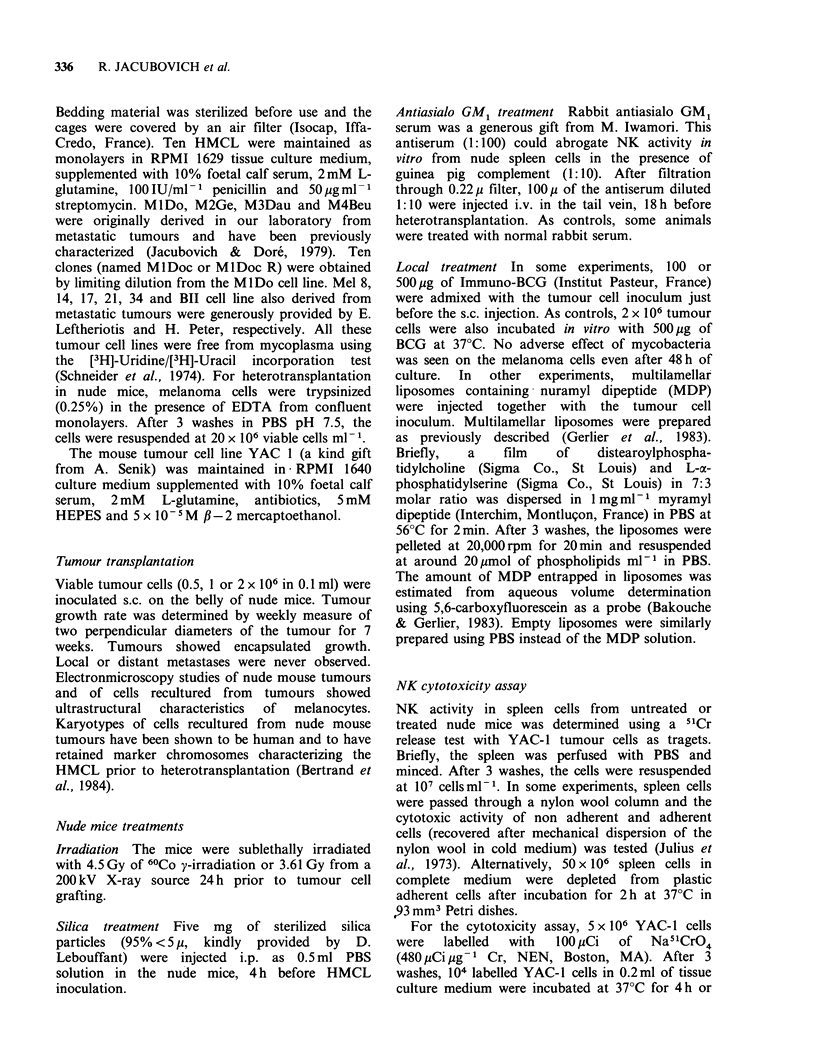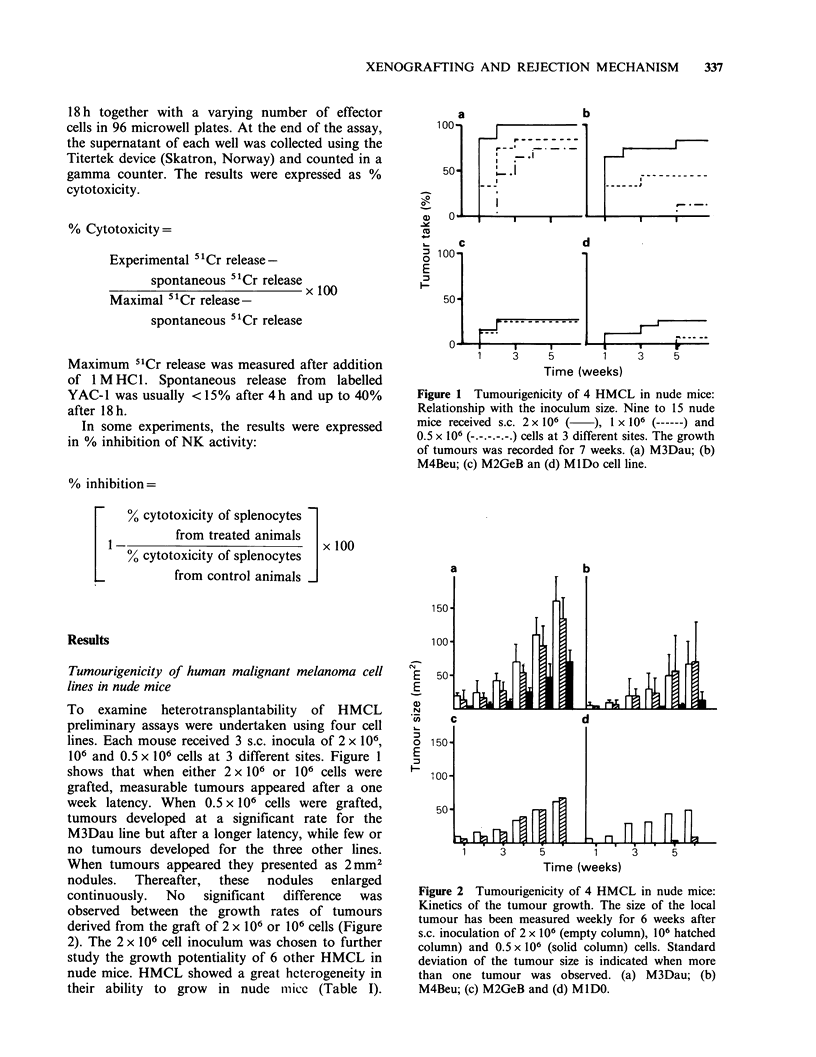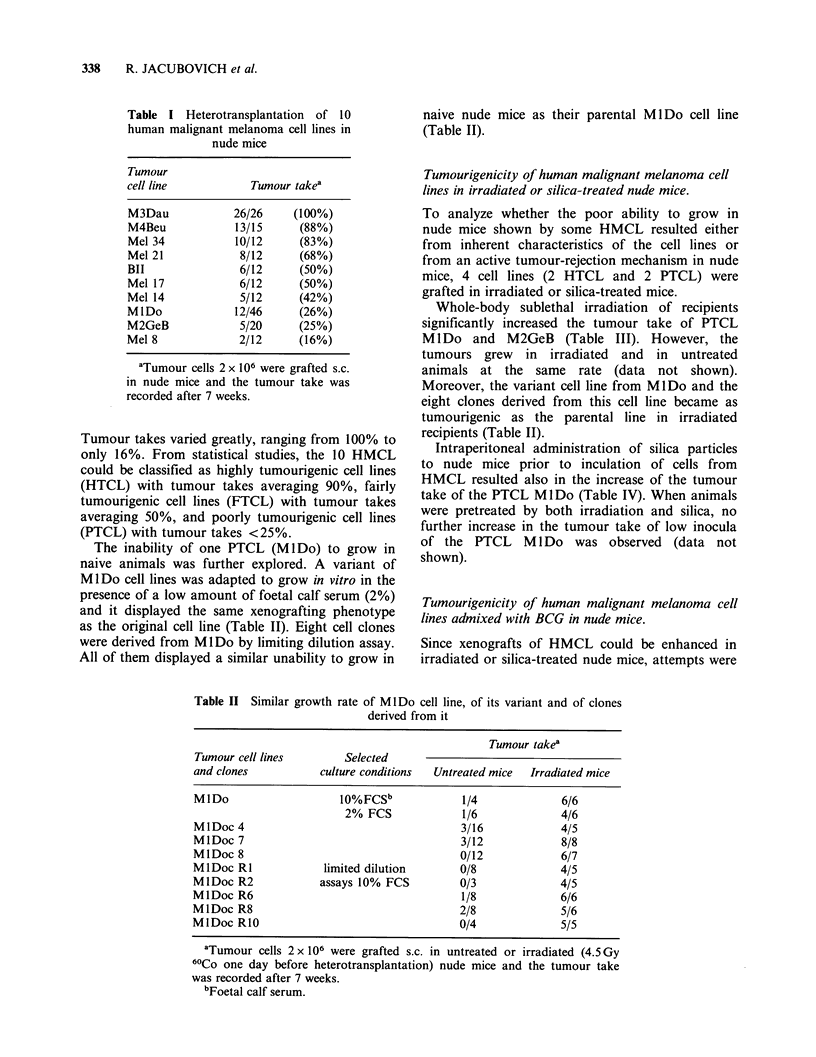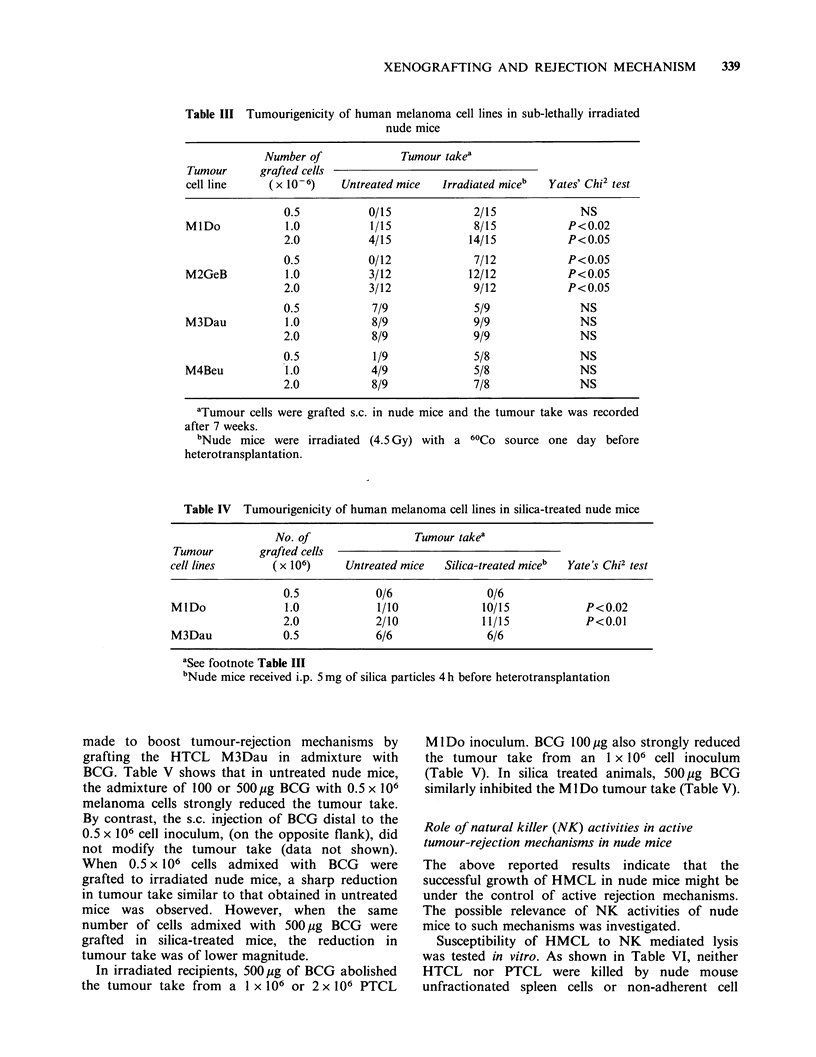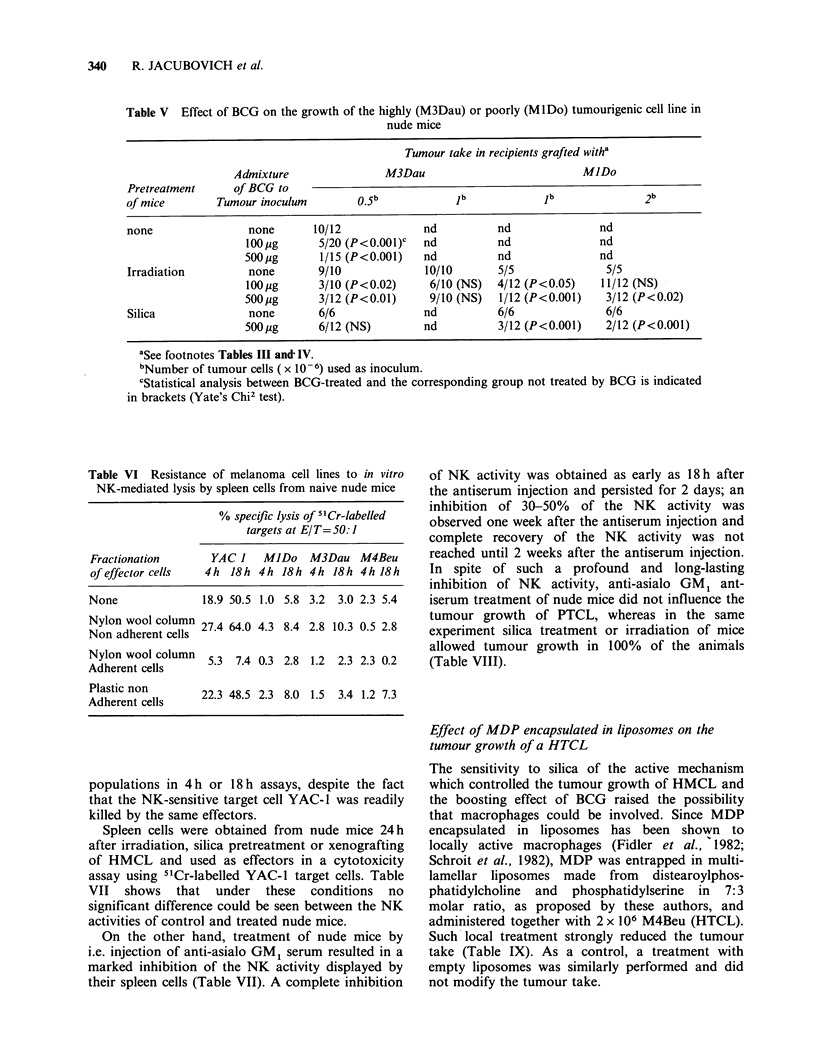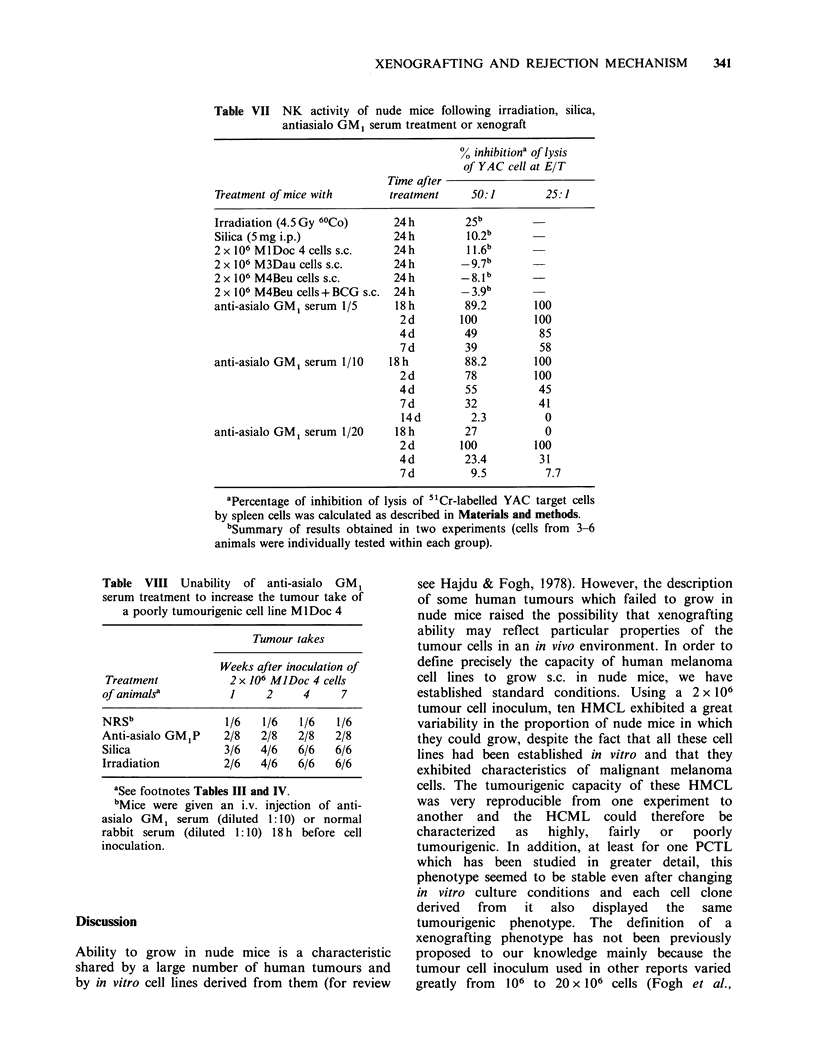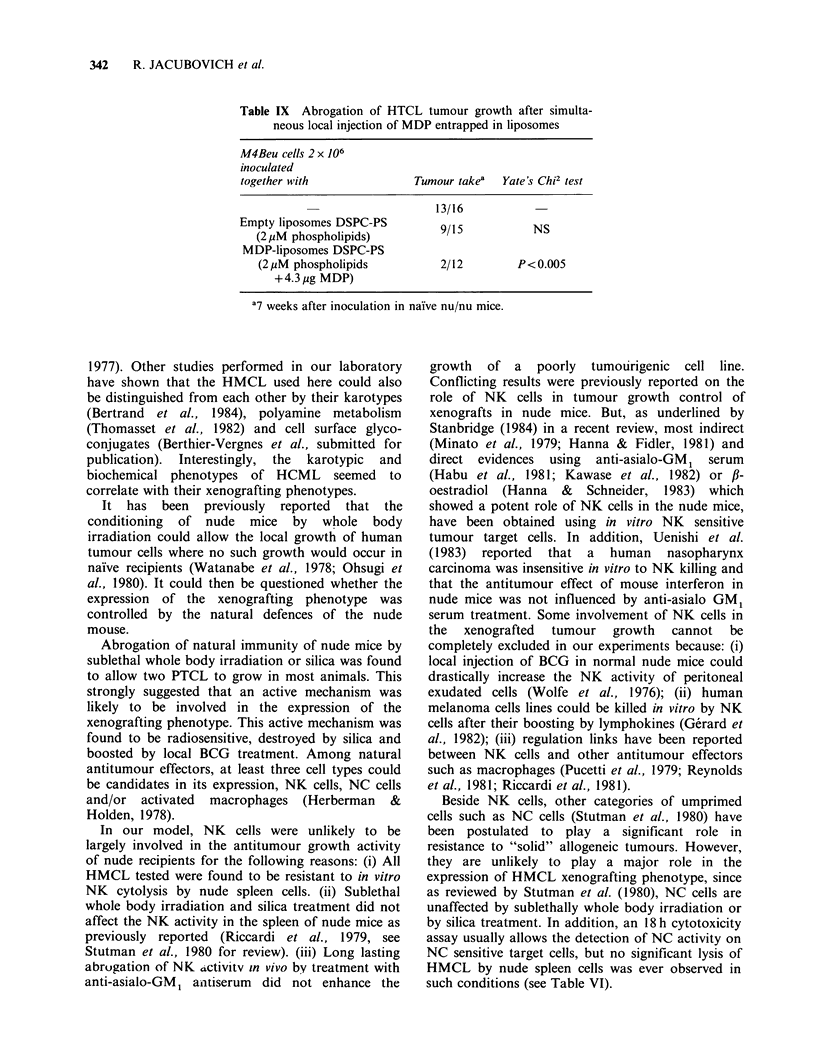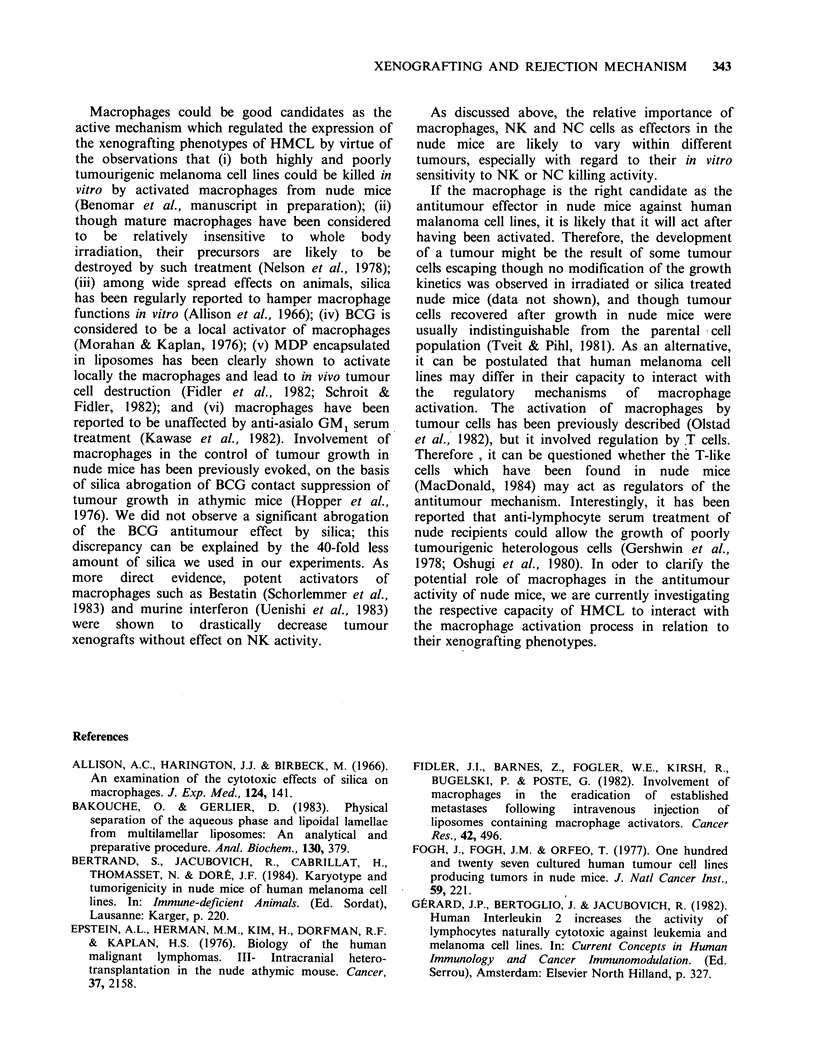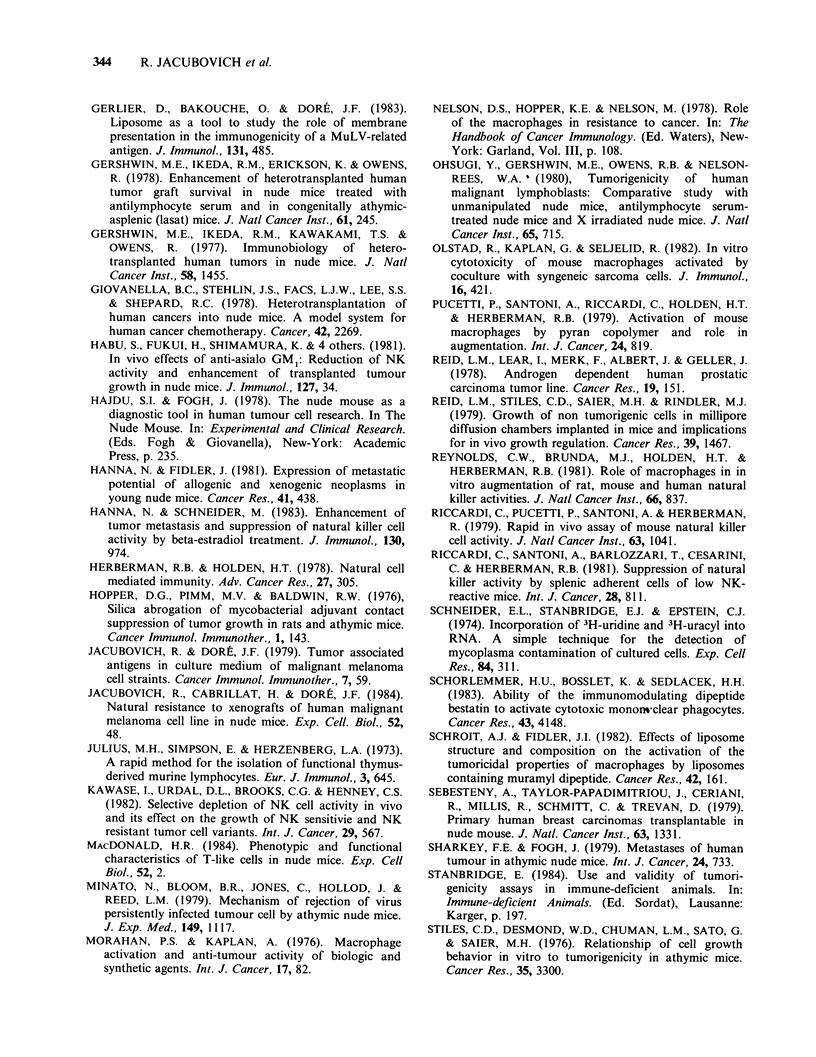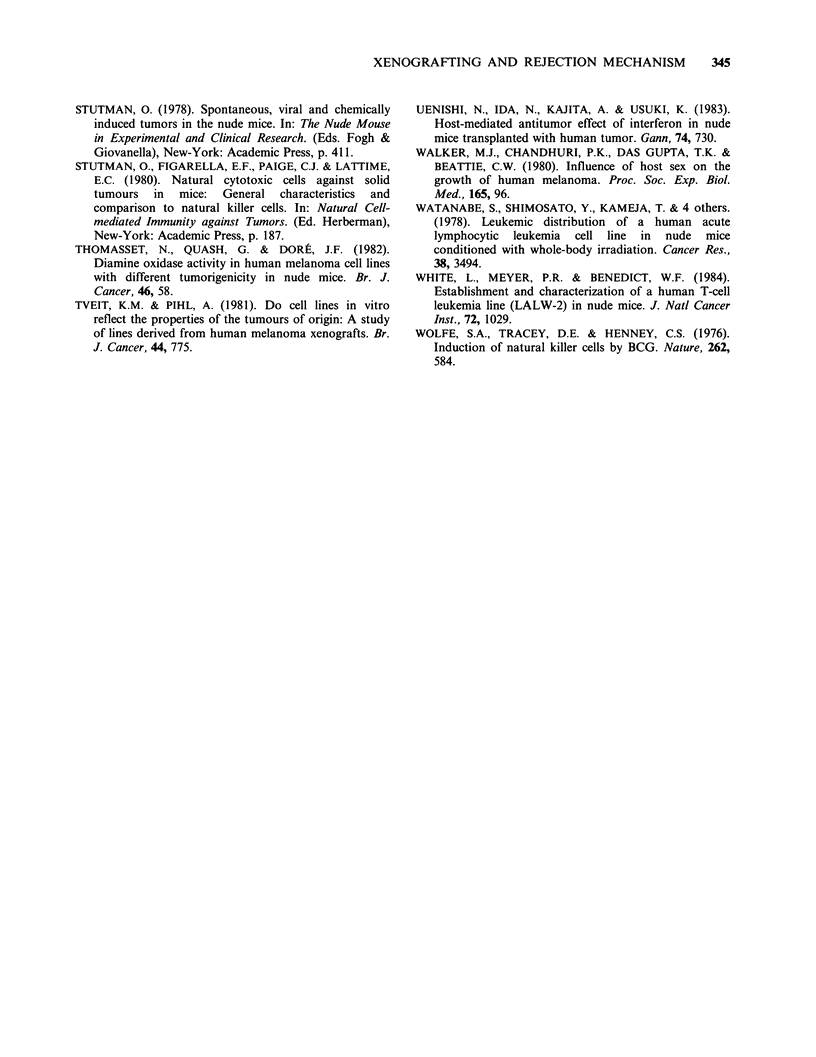# Tumourigenic phenotypes of human melanoma cell lines in nude mice determined by an active antitumour mechanism.

**DOI:** 10.1038/bjc.1985.45

**Published:** 1985-03

**Authors:** R. Jacubovich, H. Cabrillat, D. Gerlier, M. Bailly, J. F. Doré

## Abstract

Ten human melanoma cell lines (HMCL) were tested for their ability to grow subcutaneously in nude mice. Using a standard inoculum, the HMCL could be characterized by their highly, fairly or poorly xenografting phenotype. These phenotypes were stable and the phenotype of one HMCL was recovered within cell clones derived from it. The role of nude mice natural defences in the expression of HMCL xenografting phenotypes was studied. Sublethal whole body irradiation and silica pretreatment of recipients enabled poorly tumourigenic HMCL to grow in most animals without affecting their splenic NK activity. Admixture of BCG or MDP encapsulated in liposomes with highly tumourigenic HMCL resulted in the abrogation of tumour growth in naive nude mice. The long lasting abrogating of NK activity in vivo by treatment with anti-asialo-GM1 anti-serum did not enhance the growth of a poorly tumourigenic HMCL. The HMCL were found to be resistant to in vitro murine NK activity. These results showed that the expression of the HMCL xenografting phenotypes could be controlled by the nude mice natural defences. NK cells did not seem to be largely involved whereas macrophages might be good candidates as anti-xenograft effectors.


*Br. J. Cancer* (1985), **51**, 335–345

### Tumourigenic phenotypes of human melanoma cell lines in nude mice determined by an active antitumour mechanism

R. Jacubovich, H. Cabrillat, D. Gerlier, M. Bailly & J.F. Doré

*Inserum U. 218, Centre Léon Bérard, 69373 Lyon Cedex 2, France.*

**Summary** Ten human melanoma cell lines (HMCL) were tested for their ability to grow subcutaneously in nude mice. Using a standard inoculum, the HMCL could be characterized by their highly, fairly or poorly xenografting phenotype. These phenotypes were stable and the phenotype of one HMCL was recovered within cell clones derived from it. The role of nude mice natural defences in the expression of HMCL xenografting phenotypes was studied. Sublethal whole body irradiation and silica pretreatment of recipients enabled poorly tumourigenic HMCL to grow in most animals without affecting their splenic NK activity. Admixture of BCG or MDP encapsulated in liposomes with highly tumourigenic HMCL resulted in the abrogation of tumour growth in naive nude mice. The long lasting abrogating of NK activity *in vivo* by treatment with anti-asialo-$GM_1$ anti-serum did not enhance the growth of a poorly tumourigenic HMCL. The HMCL were found to be resistant to *in vitro* murine NK activity. These results showed that the expression of the HMCL xenografting phenotypes could be controlled by the nude mice natural defences. NK cells did not seem to be largely involved whereas macrophages might be good candidates as anti-xenograft effectors.

There have been numerous reports of the successful transplantation of both primary and tissue culture-passaged xenogeneic tumour cell lines into congenitally athymic (nude) mice (Fogh *et al.*, 1977; Gershwin *et al.*, 1977; Giovanella *et al.*, 1978). Initially, tumourigenicity in the nude mouse has been claimed to be a major characteristic of malignancy for primary or cultured tumour cells (Stiles *et al.*, 1976). However, some human malignant cells fail to grow in the nude mouse; among these are breast carcinoma cells (Sebesteny *et al.*, 1979), prostatic carcinoma cells (Reid *et al.*, 1978) and haemopoietic cell lines (Epstein *et al.*, 1976; White *et al.*, 1984). Here, it might be questioned whether the xenografting ability might reflect particular properties of the malignant cells in an *in vivo* environment. Numerous explanations, including substrate, local nutritional, vascular, endocrine and individual tumour specific requirements, have been proposed for the failure of some tumour lines to grow on hetero-transplantation (Reid *et al.*, 1979; Walker *et al.*, 1980). In addition, nude mice appear not to be totally immunodeficient; they do not have a higher incidence of spontaneous tumours than normal mice, are not more susceptible to chemical carcinogenesis (Stutman, 1978) and show infrequent metastases of tumours known to be metastatic in their original host (Sharkey & Fogh, 1979). The transplantation success rate of tumour growth can be enhanced by using newborn, X-irradiated or antilymphocyte serum-treated nude mice (Ohsugi *et al.*, 1980), or congenitally athymic asplenic (Lasat) mice (Gershwin *et al.*, 1978). Thus, it has been suggested that an active rejection mechanism such as natural immunity may hamper the growth of heterologous tumour cells in nude mice (Minato *et al.*, 1979).

The present studies were initiated to develop an appropriate model system for defining the xenografting phenotype of 10 human melanoma cell lines (HMCL) and for studying the possible role of the natural immunity in the tumour rejection of these cell lines by the nude mice. Using standard conditions, i.e. the same inoculum of cells, HMCL could be characterized by their xenografting phenotype which varied from highly to poorly tumourigenic, despite the fact that each of these cell lines expressed other characteristics of malignancy. Xenograft experiments in nude mice treated with agents known to depress or to stimulate natural immunity indicated that active mechanisms may be involved in the growth control of the xenogenic tumour cell lines. A major role of NK cells in xenograft rejection was not found which confirmed our preliminary results (Jacubovich *et al.*, 1984). Data are also presented here which indicated that macrophages may be involved in the xenograft rejection.

### Materials and methods

### *Animals and tumour*

Six weeks week-old male outbred Swiss nu/nu mice were purchased from IFFA-CREDO (France).

Correspondence: D. Gerlier.
Received 15 August 1984; and in revised form 13 November 1984.

© The Macmillan Press Ltd., 1985

Bedding material was sterilized before use and the cages were covered by an air filter (Isocap, Iffa-Credo, France). Ten HMCL were maintained as monolayers in RPMI 1629 tissue culture medium, supplemented with 10% foetal calf serum, 2 mM L-glutamine, 100 IU/ml$^{-1}$ penicillin and 50 $\mu$g ml$^{-1}$ streptomycin. M1Do, M2Ge, M3Dau and M4Beu were originally derived in our laboratory from metastatic tumours and have been previously characterized (Jacubovich & Doré, 1979). Ten clones (named M1Doc or M1Doc R) were obtained by limiting dilution from the M1Do cell line. Mel 8, 14, 17, 21, 34 and BII cell line also derived from metastatic tumours were generously provided by E. Leftheriotis and H. Peter, respectively. All these tumour cell lines were free from mycoplasma using the [$^3$H]-Uridine/[$^3$H]-Uracil incorporation test (Schneider et al., 1974). For heterotransplantation in nude mice, melanoma cells were trypsinized (0.25%) in the presence of EDTA from confluent monolayers. After 3 washes in PBS pH 7.5, the cells were resuspended at $20 \times 10^6$ viable cells ml$^{-1}$.

The mouse tumour cell line YAC 1 (a kind gift from A. Senik) was maintained in RPMI 1640 culture medium supplemented with 10% foetal calf serum, 2 mM L-glutamine, antibiotics, 5 mM HEPES and $5 \times 10^{-5}$ M $\beta-2$ mercaptoethanol.

*Tumour transplantation*

Viable tumour cells (0.5, 1 or $2 \times 10^6$ in 0.1 ml) were inoculated s.c. on the belly of nude mice. Tumour growth rate was determined by weekly measure of two perpendicular diameters of the tumour for 7 weeks. Tumours showed encapsulated growth. Local or distant metastases were never observed. Electronmicroscopy studies of nude mouse tumours and of cells recultured from tumours showed ultrastructural characteristics of melanocytes. Karyotypes of cells recultured from nude mouse tumours have been shown to be human and to have retained marker chromosomes characterizing the HMCL prior to heterotransplantation (Bertrand et al., 1984).

*Nude mice treatments*

*Irradiation* The mice were sublethally irradiated with 4.5 Gy of $^{60}$Co $\gamma$-irradiation or 3.61 Gy from a 200 kV X-ray source 24 h prior to tumour cell grafting.

*Silica treatment* Five mg of sterilized silica particles (95% $< 5 \mu$, kindly provided by D. Lebouffant) were injected i.p. as 0.5 ml PBS solution in the nude mice, 4 h before HMCL inoculation.

*Antiasialo $GM_1$ treatment* Rabbit antiasialo $GM_1$ serum was a generous gift from M. Iwamori. This antiserum (1:100) could abrogate NK activity *in vitro* from nude spleen cells in the presence of guinea pig complement (1:10). After filtration through 0.22 $\mu$ filter, 100 $\mu$ of the antiserum diluted 1:10 were injected i.v. in the tail vein, 18 h before heterotransplantation. As controls, some animals were treated with normal rabbit serum.

*Local treatment* In some experiments, 100 or 500 $\mu$g of Immuno-BCG (Institut Pasteur, France) were admixed with the tumour cell inoculum just before the s.c. injection. As controls, $2 \times 10^6$ tumour cells were also incubated *in vitro* with 500 $\mu$g of BCG at 37°C. No adverse effect of mycobacteria was seen on the melanoma cells even after 48 h of culture. In other experiments, multilamellar liposomes containing nuramyl dipeptide (MDP) were injected together with the tumour cell inoculum. Multilamellar liposomes were prepared as previously described (Gerlier et al., 1983). Briefly, a film of distearoylphosphatidylcholine (Sigma Co., St Louis) and L-$\alpha$-phosphatidylserine (Sigma Co., St Louis) in 7:3 molar ratio was dispersed in 1 mg ml$^{-1}$ myramyl dipeptide (Interchim, Montluçon, France) in PBS at 56°C for 2 min. After 3 washes, the liposomes were pelleted at 20,000 rpm for 20 min and resuspended at around 20 $\mu$mol of phospholipids ml$^{-1}$ in PBS. The amount of MDP entrapped in liposomes was estimated from aqueous volume determination using 5,6-carboxyfluorescein as a probe (Bakouche & Gerlier, 1983). Empty liposomes were similarly prepared using PBS instead of the MDP solution.

*NK cytotoxicity assay*

NK activity in spleen cells from untreated or treated nude mice was determined using a $^{51}$Cr release test with YAC-1 tumour cells as tragets. Briefly, the spleen was perfused with PBS and minced. After 3 washes, the cells were resuspended at $10^7$ cells ml$^{-1}$. In some experiments, spleen cells were passed through a nylon wool column and the cytotoxic activity of non adherent and adherent cells (recovered after mechanical dispersion of the nylon wool in cold medium) was tested (Julius et al., 1973). Alternatively, $50 \times 10^6$ spleen cells in complete medium were depleted from plastic adherent cells after incubation for 2 h at 37°C in 93 mm$^3$ Petri dishes.

For the cytotoxicity assay, $5 \times 10^6$ YAC-1 cells were labelled with 100 $\mu$Ci of $Na^{51}CrO_4$ (480 $\mu$Ci $\mu$g$^{-1}$ Cr, NEN, Boston, MA). After 3 washes, $10^4$ labelled YAC-1 cells in 0.2 ml of tissue culture medium were incubated at 37°C for 4 h or

18 h together with a varying number of effector cells in 96 microwell plates. At the end of the assay, the supernatant of each well was collected using the Titertek device (Skatron, Norway) and counted in a gamma counter. The results were expressed as % cytotoxicity.

$$\% \text{ Cytotoxicity} = \frac{\text{Experimental } ^{51}\text{Cr release} - \text{spontaneous } ^{51}\text{Cr release}}{\text{Maximal } ^{51}\text{Cr release} - \text{spontaneous } ^{51}\text{Cr release}} \times 100$$

Maximum $^{51}$Cr release was measured after addition of 1 M HCl. Spontaneous release from labelled YAC-1 was usually $<15\%$ after 4 h and up to 40% after 18 h.

In some experiments, the results were expressed in % inhibition of NK activity:

$$\% \text{ inhibition} = \left[1 - \frac{\% \text{ cytotoxicity of splenocytes from treated animals}}{\% \text{ cytotoxicity of splenocytes from control animals}}\right] \times 100$$

### Results

### *Tumourigenicity of human malignant melanoma cell lines in nude mice*

To examine heterotransplantability of HMCL preliminary assays were undertaken using four cell lines. Each mouse received 3 s.c. inocula of $2 \times 10^6$, $10^6$ and $0.5 \times 10^6$ cells at 3 different sites. Figure 1 shows that when either $2 \times 10^6$ or $10^6$ cells were grafted, measurable tumours appeared after a one week latency. When $0.5 \times 10^6$ cells were grafted, tumours developed at a significant rate for the M3Dau line but after a longer latency, while few or no tumours developed for the three other lines. When tumours appeared they presented as 2 mm$^2$ nodules. Thereafter, these nodules enlarged continuously. No significant difference was observed between the growth rates of tumours derived from the graft of $2 \times 10^6$ or $10^6$ cells (Figure 2). The $2 \times 10^6$ cell inoculum was chosen to further study the growth potentiality of 6 other HMCL in nude mice. HMCL showed a great heterogeneity in their ability to grow in nude mice (Table I).

**Figure 1** Tumourigenicity of 4 HMCL in nude mice: Relationship with the inoculum size. Nine to 15 nude mice received s.c. $2 \times 10^6$ (——), $1 \times 10^6$ (------) and $0.5 \times 10^6$ (-.-.-.-.-.) cells at 3 different sites. The growth of tumours was recorded for 7 weeks. (a) M3Dau; (b) M4Beu; (c) M2GeB an (d) M1Do cell line.

**Figure 2** Tumourigenicity of 4 HMCL in nude mice: Kinetics of the tumour growth. The size of the local tumour has been measured weekly for 6 weeks after s.c. inoculation of $2 \times 10^6$ (empty column), $10^6$ hatched column) and $0.5 \times 10^6$ (solid column) cells. Standard deviation of the tumour size is indicated when more than one tumour was observed. (a) M3Dau; (b) M4Beu; (c) M2GeB and (d) M1D0.

**Table I** Heterotransplantation of 10 human malignant melanoma cell lines in nude mice

| *Tumour cell line* | *Tumour take*[a] | |
|---|---|---|
| M3Dau | 26/26 | (100%) |
| M4Beu | 13/15 | (88%) |
| Mel 34 | 10/12 | (83%) |
| Mel 21 | 8/12 | (68%) |
| BII | 6/12 | (50%) |
| Mel 17 | 6/12 | (50%) |
| Mel 14 | 5/12 | (42%) |
| M1Do | 12/46 | (26%) |
| M2GeB | 5/20 | (25%) |
| Mel 8 | 2/12 | (16%) |

[a]Tumour cells $2 \times 10^6$ were grafted s.c. in nude mice and the tumour take was recorded after 7 weeks.

Tumour takes varied greatly, ranging from 100% to only 16%. From statistical studies, the 10 HMCL could be classified as highly tumourigenic cell lines (HTCL) with tumour takes averaging 90%, fairly tumourigenic cell lines (FTCL) with tumour takes averaging 50%, and poorly tumourigenic cell lines (PTCL) with tumour takes <25%.

The inability of one PTCL (M1Do) to grow in naive animals was further explored. A variant of M1Do cell lines was adapted to grow *in vitro* in the presence of a low amount of foetal calf serum (2%) and it displayed the same xenografting phenotype as the original cell line (Table II). Eight cell clones were derived from M1Do by limiting dilution assay. All of them displayed a similar unability to grow in naive nude mice as their parental M1Do cell line (Table II).

*Tumourigenicity of human malignant melanoma cell lines in irradiated or silica-treated nude mice.*

To analyze whether the poor ability to grow in nude mice shown by some HMCL resulted either from inherent characteristics of the cell lines or from an active tumour-rejection mechanism in nude mice, 4 cell lines (2 HTCL and 2 PTCL) were grafted in irradiated or silica-treated mice.

Whole-body sublethal irradiation of recipients significantly increased the tumour take of PTCL M1Do and M2GeB (Table III). However, the tumours grew in irradiated and in untreated animals at the same rate (data not shown). Moreover, the variant cell line from M1Do and the eight clones derived from this cell line became as tumourigenic as the parental line in irradiated recipients (Table II).

Intraperitoneal administration of silica particles to nude mice prior to inculation of cells from HMCL resulted also in the increase of the tumour take of the PTCL M1Do (Table IV). When animals were pretreated by both irradiation and silica, no further increase in the tumour take of low inocula of the PTCL M1Do was observed (data not shown).

*Tumourigenicity of human malignant melanoma cell lines admixed with BCG in nude mice.*

Since xenografts of HMCL could be enhanced in irradiated or silica-treated nude mice, attempts were

**Table II** Similar growth rate of M1Do cell line, of its variant and of clones derived from it

| *Tumour cell lines and clones* | *Selected culture conditions* | *Tumour take*[a] | |
|---|---|---|---|
| | | *Untreated mice* | *Irradiated mice* |
| M1Do | 10%FCS[b] | 1/4 | 6/6 |
| | 2% FCS | 1/6 | 4/6 |
| M1Doc 4 | | 3/16 | 4/5 |
| M1Doc 7 | | 3/12 | 8/8 |
| M1Doc 8 | | 0/12 | 6/7 |
| M1Doc R1 | limited dilution | 0/8 | 4/5 |
| M1Doc R2 | assays 10% FCS | 0/3 | 4/5 |
| M1Doc R6 | | 1/8 | 6/6 |
| M1Doc R8 | | 2/8 | 5/6 |
| M1Doc R10 | | 0/4 | 5/5 |

[a]Tumour cells $2 \times 10^6$ were grafted s.c. in untreated or irradiated (4.5 Gy $^{60}Co$ one day before heterotransplantation) nude mice and the tumour take was recorded after 7 weeks.

[b]Foetal calf serum.

**Table III** Tumourigenicity of human melanoma cell lines in sub-lethally irradiated nude mice

| Tumour cell line | Number of grafted cells ($\times 10^{-6}$) | *Tumour take*[a] | | *Yates' Chi*$^2$ *test* |
|---|---|---|---|---|
| | | *Untreated mice* | *Irradiated mice*[b] | |
| | 0.5 | 0/15 | 2/15 | NS |
| M1Do | 1.0 | 1/15 | 8/15 | $P<0.02$ |
| | 2.0 | 4/15 | 14/15 | $P<0.05$ |
| | 0.5 | 0/12 | 7/12 | $P<0.05$ |
| M2GeB | 1.0 | 3/12 | 12/12 | $P<0.05$ |
| | 2.0 | 3/12 | 9/12 | $P<0.05$ |
| | 0.5 | 7/9 | 5/9 | NS |
| M3Dau | 1.0 | 8/9 | 9/9 | NS |
| | 2.0 | 8/9 | 9/9 | NS |
| | 0.5 | 1/9 | 5/8 | NS |
| M4Beu | 1.0 | 4/9 | 5/8 | NS |
| | 2.0 | 8/9 | 7/8 | NS |

[a]Tumour cells were grafted s.c. in nude mice and the tumour take was recorded after 7 weeks.

[b]Nude mice were irradiated (4.5 Gy) with a $^{60}Co$ source one day before heterotransplantation.

**Table IV** Tumourigenicity of human melanoma cell lines in silica-treated nude mice

| *Tumour cell lines* | *No. of grafted cells* ($\times 10^{6}$) | *Tumour take*[a] | | *Yate's Chi*$^2$ *test* |
|---|---|---|---|---|
| | | *Untreated mice* | *Silica-treated mice*[b] | |
| | 0.5 | 0/6 | 0/6 | |
| M1Do | 1.0 | 1/10 | 10/15 | $P<0.02$ |
| | 2.0 | 2/10 | 11/15 | $P<0.01$ |
| M3Dau | 0.5 | 6/6 | 6/6 | |

[a]See footnote **Table III**

[b]Nude mice received i.p. 5 mg of silica particles 4 h before heterotransplantation made to boost tumour-rejection mechanisms by grafting the HTCL M3Dau in admixture with BCG. Table V shows that in untreated nude mice, the admixture of 100 or 500 $\mu$g BCG with $0.5 \times 10^6$ melanoma cells strongly reduced the tumour take. By contrast, the s.c. injection of BCG distal to the $0.5 \times 10^6$ cell inoculum, (on the opposite flank), did not modify the tumour take (data not shown). When $0.5 \times 10^6$ cells admixed with BCG were grafted to irradiated nude mice, a sharp reduction in tumour take similar to that obtained in untreated mice was observed. However, when the same number of cells admixed with 500 $\mu$g BCG were grafted in silica-treated mice, the reduction in tumour take was of lower magnitude.

In irradiated recipients, 500 $\mu$g of BCG abolished the tumour take from a $1 \times 10^6$ or $2 \times 10^6$ PTCL M1Do inoculum. BCG 100 $\mu$g also strongly reduced the tumour take from an $1 \times 10^6$ cell inoculum (Table V). In silica treated animals, 500 $\mu$g BCG similarly inhibited the M1Do tumour take (Table V).

### *Role of natural killer (NK) activities in active tumour-rejection mechanisms in nude mice*

The above reported results indicate that the successful growth of HMCL in nude mice might be under the control of active rejection mechanisms. The possible relevance of NK activities of nude mice to such mechanisms was investigated.

Susceptibility of HMCL to NK mediated lysis was tested *in vitro*. As shown in Table VI, neither HTCL nor PTCL were killed by nude mouse unfractionated spleen cells or non-adherent cell

**Table V** Effect of BCG on the growth of the highly (M3Dau) or poorly (M1Do) tumourigenic cell line in nude mice

| *Pretreatment of mice* | *Admixture of BCG to Tumour inoculum* | *Tumour take in recipients grafted with*[a] *M3Dau* *0.5*[b] | *M3Dau* *1*[b] | *M1Do* *1*[b] | *M1Do* *2*[b] |
|---|---|---|---|---|---|
| none | none | 10/12 | nd | nd | nd |
| | 100 μg | 5/20 ($P$<0.001)[c] | nd | nd | nd |
| | 500 μg | 1/15 ($P$<0.001) | nd | nd | nd |
| Irradiation | none | 9/10 | 10/10 | 5/5 | 5/5 |
| | 100 μg | 3/10 ($P$<0.02) | 6/10 (NS) | 4/12 ($P$<0.05) | 11/12 (NS) |
| | 500 μg | 3/12 ($P$<0.01) | 9/10 (NS) | 1/12 ($P$<0.001) | 3/12 ($P$<0.02) |
| Silica | none | 6/6 | nd | 6/6 | 6/6 |
| | 500 μg | 6/12 (NS) | nd | 3/12 ($P$<0.001) | 2/12 ($P$<0.001) |

[a]See footnotes **Tables III and IV**.
[b]Number of tumour cells ( $\times 10^{-6}$) used as inoculum.
[c]Statistical analysis between BCG-treated and the corresponding group not treated by BCG is indicated in brackets (Yate's Chi$^2$ test).

**Table VI** Resistance of melanoma cell lines to *in vitro* NK-mediated lysis by spleen cells from naive nude mice

| *Fractionation of effector cells* | *% specific lysis of* $^{51}$*Cr-labelled targets at E/T=50:1* *YAC 1* *4h* | *YAC 1* *18h* | *M1Do* *4h* | *M1Do* *18h* | *M3Dau* *4h* | *M3Dau* *18h* | *M4Beu* *4h* | *M4Beu* *18h* |
|---|---|---|---|---|---|---|---|---|
| None | 18.9 | 50.5 | 1.0 | 5.8 | 3.2 | 3.0 | 2.3 | 5.4 |
| Nylon wool column Non adherent cells | 27.4 | 64.0 | 4.3 | 8.4 | 2.8 | 10.3 | 0.5 | 2.8 |
| Nylon wool column Adherent cells | 5.3 | 7.4 | 0.3 | 2.8 | 1.2 | 2.3 | 2.3 | 0.2 |
| Plastic non Adherent cells | 22.3 | 48.5 | 2.3 | 8.0 | 1.5 | 3.4 | 1.2 | 7.3 |

populations in 4 h or 18 h assays, despite the fact that the NK-sensitive target cell YAC-1 was readily killed by the same effectors.

Spleen cells were obtained from nude mice 24 h after irradiation, silica pretreatment or xenografting of HMCL and used as effectors in a cytotoxicity assay using $^{51}$Cr-labelled YAC-1 target cells. Table VII shows that under these conditions no significant difference could be seen between the NK activities of control and treated nude mice.

On the other hand, treatment of nude mice by i.e. injection of anti-asialo GM$_1$ serum resulted in a marked inhibition of the NK activity displayed by their spleen cells (Table VII). A complete inhibition of NK activity was obtained as early as 18 h after the antiserum injection and persisted for 2 days; an inhibition of 30–50% of the NK activity was observed one week after the antiserum injection and complete recovery of the NK activity was not reached until 2 weeks after the antiserum injection. In spite of such a profound and long-lasting inhibition of NK activity, anti-asialo GM$_1$ antiserum treatment of nude mice did not influence the tumour growth of PTCL, whereas in the same experiment silica treatment or irradiation of mice allowed tumour growth in 100% of the animals (Table VIII).

*Effect of MDP encapsulated in liposomes on the tumour growth of a HTCL*

The sensitivity to silica of the active mechanism which controlled the tumour growth of HMCL and the boosting effect of BCG raised the possibility that macrophages could be involved. Since MDP encapsulated in liposomes has been shown to locally active macrophages (Fidler *et al.*, 1982; Schroit *et al.*, 1982), MDP was entrapped in multilamellar liposomes made from distearoylphosphatidylcholine and phosphatidylserine in 7:3 molar ratio, as proposed by these authors, and administered together with $2\times10^6$ M4Beu (HTCL). Such local treatment strongly reduced the tumour take (Table IX). As a control, a treatment with empty liposomes was similarly performed and did not modify the tumour take.

**Table VII** NK activity of nude mice following irradiation, silica, antiasialo $GM_1$ serum treatment or xenograft

| *Treatment of mice with* | *Time after treatment* | *% inhibition*[a] *of lysis of YAC cell at E/T* 50:1 | 25:1 |
|---|---|---|---|
| Irradiation (4.5 Gy $^{60}Co$) | 24 h | 25[b] | — |
| Silica (5 mg i.p.) | 24 h | 10.2[b] | — |
| $2\times10^6$ M1Doc 4 cells s.c. | 24 h | 11.6[b] | — |
| $2\times10^6$ M3Dau cells s.c. | 24 h | −9.7[b] | — |
| $2\times10^6$ M4Beu cells s.c. | 24 h | −8.1[b] | — |
| $2\times10^6$ M4Beu cells + BCG s.c. | 24 h | −3.9[b] | — |
| anti-asialo $GM_1$ serum 1/5 | 18 h | 89.2 | 100 |
| | 2 d | 100 | 100 |
| | 4 d | 49 | 85 |
| | 7 d | 39 | 58 |
| anti-asialo $GM_1$ serum 1/10 | 18 h | 88.2 | 100 |
| | 2 d | 78 | 100 |
| | 4 d | 55 | 45 |
| | 7 d | 32 | 41 |
| | 14 d | 2.3 | 0 |
| anti-asialo $GM_1$ serum 1/20 | 18 h | 27 | 0 |
| | 2 d | 100 | 100 |
| | 4 d | 23.4 | 31 |
| | 7 d | 9.5 | 7.7 |

[a]Percentage of inhibition of lysis of $^{51}Cr$-labelled YAC target cells by spleen cells was calculated as described in **Materials and methods**.
[b]Summary of results obtained in two experiments (cells from 3–6 animals were individually tested within each group).

**Table VIII** Unability of anti-asialo $GM_1$ serum treatment to increase the tumour take of a poorly tumourigenic cell line M1Doc 4

| *Treatment of animals*[a] | *Tumour takes* — *Weeks after inoculation of $2\times10^6$ M1Doc 4 cells* 1 | 2 | 4 | 7 |
|---|---|---|---|---|
| NRS[b] | 1/6 | 1/6 | 1/6 | 1/6 |
| Anti-asialo $GM_1P$ | 2/8 | 2/8 | 2/8 | 2/8 |
| Silica | 3/6 | 4/6 | 6/6 | 6/6 |
| Irradiation | 2/6 | 4/6 | 6/6 | 6/6 |

[a]See footnotes **Tables III and IV**.
[b]Mice were given an i.v. injection of anti-asialo $GM_1$ serum (diluted 1:10) or normal rabbit serum (diluted 1:10) 18 h before cell inoculation.

### Discussion

Ability to grow in nude mice is a characteristic shared by a large number of human tumours and by *in vitro* cell lines derived from them (for review see Hajdu & Fogh, 1978). However, the description of some human tumours which failed to grow in nude mice raised the possibility that xenografting ability may reflect particular properties of the tumour cells in an *in vivo* environment. In order to define precisely the capacity of human melanoma cell lines to grow s.c. in nude mice, we have established standard conditions. Using a $2\times10^6$ tumour cell inoculum, ten HMCL exhibited a great variability in the proportion of nude mice in which they could grow, despite the fact that all these cell lines had been established *in vitro* and that they exhibited characteristics of malignant melanoma cells. The tumourigenic capacity of these HMCL was very reproducible from one experiment to another and the HCML could therefore be characterized as highly, fairly or poorly tumourigenic. In addition, at least for one PCTL which has been studied in greater detail, this phenotype seemed to be stable even after changing *in vitro* culture conditions and each cell clone derived from it also displayed the same tumourigenic phenotype. The definition of a xenografting phenotype has not been previously proposed to our knowledge mainly because the tumour cell inoculum used in other reports varied greatly from $10^6$ to $20\times10^6$ cells (Fogh *et al.*,

**Table IX** Abrogation of HTCL tumour growth after simultaneous local injection of MDP entrapped in liposomes

| *M4Beu cells* $2\times 10^6$ *inoculated together with* | *Tumour take*[a] | *Yate's* $Chi^2$ *test* |
|---|---|---|
| — | 13/16 | — |
| Empty liposomes DSPC-PS (2 $\mu$M phospholipids) | 9/15 | NS |
| MDP-liposomes DSPC-PS (2 $\mu$M phospholipids + 4.3 $\mu$g MDP) | 2/12 | $P<0.005$ |

[a]7 weeks after inoculation in naïve nu/nu mice.

1977). Other studies performed in our laboratory have shown that the HMCL used here could also be distinguished from each other by their karotypes (Bertrand *et al.*, 1984), polyamine metabolism (Thomasset *et al.*, 1982) and cell surface glycoconjugates (Berthier-Vergnes *et al.*, submitted for publication). Interestingly, the karotypic and biochemical phenotypes of HCML seemed to correlate with their xenografting phenotypes.

It has been previously reported that the conditioning of nude mice by whole body irradiation could allow the local growth of human tumour cells where no such growth would occur in naïve recipients (Watanabe *et al.*, 1978; Ohsugi *et al.*, 1980). It could then be questioned whether the expression of the xenografting phenotype was controlled by the natural defences of the nude mouse.

Abrogation of natural immunity of nude mice by sublethal whole body irradiation or silica was found to allow two PTCL to grow in most animals. This strongly suggested that an active mechanism was likely to be involved in the expression of the xenografting phenotype. This active mechanism was found to be radiosensitive, destroyed by silica and boosted by local BCG treatment. Among natural antitumour effectors, at least three cell types could be candidates in its expression, NK cells, NC cells and/or activated macrophages (Herberman & Holden, 1978).

In our model, NK cells were unlikely to be largely involved in the antitumour growth activity of nude recipients for the following reasons: (i) All HMCL tested were found to be resistant to *in vitro* NK cytolysis by nude spleen cells. (ii) Sublethal whole body irradiation and silica treatment did not affect the NK activity in the spleen of nude mice as previously reported (Riccardi *et al.*, 1979, see Stutman *et al.*, 1980 for review). (iii) Long lasting abrogation of NK activity *in vivo* by treatment with anti-asialo-$GM_1$ antiserum did not enhance the growth of a poorly tumourigenic cell line. Conflicting results were previously reported on the role of NK cells in tumour growth control of xenografts in nude mice. But, as underlined by Stanbridge (1984) in a recent review, most indirect (Minato *et al.*, 1979; Hanna & Fidler, 1981) and direct evidences using anti-asialo-$GM_1$ serum (Habu *et al.*, 1981; Kawase *et al.*, 1982) or $\beta$-oestradiol (Hanna & Schneider, 1983) which showed a potent role of NK cells in the nude mice, have been obtained using *in vitro* NK sensitive tumour target cells. In addition, Uenishi *et al.* (1983) reported that a human nasopharynx carcinoma was insensitive *in vitro* to NK killing and that the antitumour effect of mouse interferon in nude mice was not influenced by anti-asialo $GM_1$ serum treatment. Some involvement of NK cells in the xenografted tumour growth cannot be completely excluded in our experiments because: (i) local injection of BCG in normal nude mice could drastically increase the NK activity of peritoneal exudated cells (Wolfe *et al.*, 1976); (ii) human melanoma cells lines could be killed *in vitro* by NK cells after their boosting by lymphokines (Gérard *et al.*, 1982); (iii) regulation links have been reported between NK cells and other antitumour effectors such as macrophages (Pucetti *et al.*, 1979; Reynolds *et al.*, 1981; Riccardi *et al.*, 1981).

Beside NK cells, other categories of umprimed cells such as NC cells (Stutman *et al.*, 1980) have been postulated to play a significant role in resistance to "solid" allogeneic tumours. However, they are unlikely to play a major role in the expression of HMCL xenografting phenotype, since as reviewed by Stutman *et al.* (1980), NC cells are unaffected by sublethally whole body irradiation or by silica treatment. In addition, an 18 h cytotoxicity assay usually allows the detection of NC activity on NC sensitive target cells, but no significant lysis of HMCL by nude spleen cells was ever observed in such conditions (see Table VI).

Macrophages could be good candidates as the active mechanism which regulated the expression of the xenografting phenotypes of HMCL by virtue of the observations that (i) both highly and poorly tumourigenic melanoma cell lines could be killed *in vitro* by activated macrophages from nude mice (Benomar *et al.*, manuscript in preparation); (ii) though mature macrophages have been considered to be relatively insensitive to whole body irradiation, their precursors are likely to be destroyed by such treatment (Nelson *et al.*, 1978); (iii) among wide spread effects on animals, silica has been regularly reported to hamper macrophage functions *in vitro* (Allison *et al.*, 1966); (iv) BCG is considered to be a local activator of macrophages (Morahan & Kaplan, 1976); (v) MDP encapsulated in liposomes has been clearly shown to activate locally the macrophages and lead to *in vivo* tumour cell destruction (Fidler *et al.*, 1982; Schroit & Fidler, 1982); and (vi) macrophages have been reported to be unaffected by anti-asialo $GM_1$ serum treatment (Kawase *et al.*, 1982). Involvement of macrophages in the control of tumour growth in nude mice has been previously evoked, on the basis of silica abrogation of BCG contact suppression of tumour growth in athymic mice (Hopper *et al.*, 1976). We did not observe a significant abrogation of the BCG antitumour effect by silica; this discrepancy can be explained by the 40-fold less amount of silica we used in our experiments. As more direct evidence, potent activators of macrophages such as Bestatin (Schorlemmer *et al.*, 1983) and murine interferon (Uenishi *et al.*, 1983) were shown to drastically decrease tumour xenografts without effect on NK activity.

As discussed above, the relative importance of macrophages, NK and NC cells as effectors in the nude mice are likely to vary within different tumours, especially with regard to their *in vitro* sensitivity to NK or NC killing activity.

If the macrophage is the right candidate as the antitumour effector in nude mice against human malanoma cell lines, it is likely that it will act after having been activated. Therefore, the development of a tumour might be the result of some tumour cells escaping though no modification of the growth kinetics was observed in irradiated or silica treated nude mice (data not shown), and though tumour cells recovered after growth in nude mice were usually indistinguishable from the parental cell population (Tveit & Pihl, 1981). As an alternative, it can be postulated that human melanoma cell lines may differ in their capacity to interact with the regulatory mechanisms of macrophage activation. The activation of macrophages by tumour cells has been previously described (Olstad *et al.*, 1982), but it involved regulation by T cells. Therefore , it can be questioned whether the T-like cells which have been found in nude mice (MacDonald, 1984) may act as regulators of the antitumour mechanism. Interestingly, it has been reported that anti-lymphocyte serum treatment of nude recipients could allow the growth of poorly tumourigenic heterologous cells (Gershwin *et al.*, 1978; Oshugi *et al.*, 1980). In oder to clarify the potential role of macrophages in the antitumour activity of nude mice, we are currently investigating the respective capacity of HMCL to interact with the macrophage activation process in relation to their xenografting phenotypes.

### References

ALLISON, A.C., HARINGTON, J.J. & BIRBECK, M. (1966). An examination of the cytotoxic effects of silica on macrophages. *J. Exp. Med.*, **124,** 141.

BAKOUCHE, O. & GERLIER, D. (1983). Physical separation of the aqueous phase and lipoidal lamellae from multilamellar liposomes: An analytical and preparative procedure. *Anal. Biochem.*, **130,** 379.

BERTRAND, S., JACUBOVICH, R., CABRILLAT, H., THOMASSET, N. & DORÉ, J.F. (1984). Karyotype and tumorigenicity in nude mice of human melanoma cell lines. In: *Immune-deficient Animals*. (Ed. Sordat), Lausanne: Karger, p. 220.

EPSTEIN, A.L., HERMAN, M.M., KIM, H., DORFMAN, R.F. & KAPLAN, H.S. (1976). Biology of the human malignant lymphomas. III- Intracranial hetero-transplantation in the nude athymic mouse. *Cancer*, **37,** 2158.

FIDLER, J.I., BARNES, Z., FOGLER, W.E., KIRSH, R., BUGELSKI, P. & POSTE, G. (1982). Involvement of macrophages in the eradication of established metastases following intravenous injection of liposomes containing macrophage activators. *Cancer Res.*, **42,** 496.

FOGH, J., FOGH, J.M. & ORFEO, T. (1977). One hundred and twenty seven cultured human tumour cell lines producing tumors in nude mice. *J. Natl Cancer Inst.*, **59,** 221.

GÉRARD, J.P., BERTOGLIO, J. & JACUBOVICH, R. (1982). Human Interleukin 2 increases the activity of lymphocytes naturally cytotoxic against leukemia and melanoma cell lines. In: *Current Concepts in Human Immunology and Cancer Immunomodulation*. (Ed. Serrou), Amsterdam: Elsevier North Hilland, p. 327.

GERLIER, D., BAKOUCHE, O. & DORÉ, J.F. (1983). Liposome as a tool to study the role of membrane presentation in the immunogenicity of a MuLV-related antigen. *J. Immunol.*, **131**, 485.

GERSHWIN, M.E., IKEDA, R.M., ERICKSON, K. & OWENS, R. (1978). Enhancement of heterotransplanted human tumor graft survival in nude mice treated with antilymphocyte serum and in congenitally athymic-asplenic (lasat) mice. *J. Natl Cancer Inst.*, **61**, 245.

GERSHWIN, M.E., IKEDA, R.M., KAWAKAMI, T.S. & OWENS, R. (1977). Immunobiology of heterotransplanted human tumors in nude mice. *J. Natl Cancer Inst.*, **58**, 1455.

GIOVANELLA, B.C., STEHLIN, J.S., FACS, L.J.W., LEE, S.S. & SHEPARD, R.C. (1978). Heterotransplantation of human cancers into nude mice. A model system for human cancer chemotherapy. *Cancer*, **42**, 2269.

HABU, S., FUKUI, H., SHIMAMURA, K. & 4 others. (1981). In vivo effects of anti-asialo $GM_1$: Reduction of NK activity and enhancement of transplanted tumour growth in nude mice. *J. Immunol.*, **127**, 34.

HAJDU, S.I. & FOGH, J. (1978). The nude mouse as a diagnostic tool in human tumour cell research. In The Nude Mouse. In: *Experimental and Clinical Research*. (Eds. Fogh & Giovanella), New-York: Academic Press, p. 235.

HANNA, N. & FIDLER, J. (1981). Expression of metastatic potential of allogenic and xenogenic neoplasms in young nude mice. *Cancer Res.*, **41**, 438.

HANNA, N. & SCHNEIDER, M. (1983). Enhancement of tumor metastasis and suppression of natural killer cell activity by beta-estradiol treatment. *J. Immunol.*, **130**, 974.

HERBERMAN, R.B. & HOLDEN, H.T. (1978). Natural cell mediated immunity. *Adv. Cancer Res.*, **27**, 305.

HOPPER, D.G., PIMM, M.V. & BALDWIN, R.W. (1976), Silica abrogation of mycobacterial adjuvant contact suppression of tumor growth in rats and athymic mice. *Cancer Immunol. Immunother.*, **1**, 143.

JACUBOVICH, R. & DORÉ, J.F. (1979). Tumor associated antigens in culture medium of malignant melanoma cell straints. *Cancer Immunol. Immunother.*, **7**, 59.

JACUBOVICH, R., CABRILLAT, H. & DORÉ, J.F. (1984). Natural resistance to xenografts of human malignant melanoma cell line in nude mice. *Exp. Cell. Biol.*, **52**, 48.

JULIUS, M.H., SIMPSON, E. & HERZENBERG, L.A. (1973). A rapid method for the isolation of functional thymus-derived murine lymphocytes. *Eur. J. Immunol.*, **3**, 645.

KAWASE, I., URDAL, D.L., BROOKS, C.G. & HENNEY, C.S. (1982). Selective depletion of NK cell activity in vivo and its effect on the growth of NK sensitivie and NK resistant tumor cell variants. *Int. J. Cancer*, **29**, 567.

MACDONALD, H.R. (1984). Phenotypic and functional characteristics of T-like cells in nude mice. *Exp. Cell Biol.*, **52**, 2.

MINATO, N., BLOOM, B.R., JONES, C., HOLLOD, J. & REED, L.M. (1979). Mechanism of rejection of virus persistently infected tumour cell by athymic nude mice. *J. Exp. Med.*, **149**, 1117.

MORAHAN, P.S. & KAPLAN, A. (1976). Macrophage activation and anti-tumour activity of biologic and synthetic agents. *Int. J. Cancer*, **17**, 82.

NELSON, D.S., HOPPER, K.E. & NELSON, M. (1978). Role of the macrophages in resistance to cancer. In: *The Handbook of Cancer Immunology*. (Ed. Waters), New-York: Garland, Vol. III, p. 108.

OHSUGI, Y., GERSHWIN, M.E., OWENS, R.B. & NELSON-REES, W.A. ' (1980), Tumorigenicity of human malignant lymphoblasts: Comparative study with unmanipulated nude mice, antilymphocyte serum-treated nude mice and X irradiated nude mice. *J. Natl Cancer Inst.*, **65**, 715.

OLSTAD, R., KAPLAN, G. & SELJELID, R. (1982). In vitro cytotoxicity of mouse macrophages activated by coculture with syngeneic sarcoma cells. *J. Immunol.*, **16**, 421.

PUCETTI, P., SANTONI, A., RICCARDI, C., HOLDEN, H.T. & HERBERMAN, R.B. (1979). Activation of mouse macrophages by pyran copolymer and role in augmentation. *Int. J. Cancer*, **24**, 819.

REID, L.M., LEAR, I., MERK, F., ALBERT, J. & GELLER, J. (1978). Androgen dependent human prostatic carcinoma tumor line. *Cancer Res.*, **19**, 151.

REID, L.M., STILES, C.D., SAIER, M.H. & RINDLER, M.J. (1979). Growth of non tumorigenic cells in millipore diffusion chambers implanted in mice and implications for in vivo growth regulation. *Cancer Res.*, **39**, 1467.

REYNOLDS, C.W., BRUNDA, M.J., HOLDEN, H.T. & HERBERMAN, R.B. (1981). Role of macrophages in in vitro augmentation of rat, mouse and human natural killer activities. *J. Natl Cancer Inst.*, **66**, 837.

RICCARDI, C., PUCETTI, P., SANTONI, A. & HERBERMAN, R. (1979). Rapid in vivo assay of mouse natural killer cell activity. *J. Natl Cancer Inst.*, **63**, 1041.

RICCARDI, C., SANTONI, A., BARLOZZARI, T., CESARINI, C. & HERBERMAN, R.B. (1981). Suppression of natural killer activity by splenic adherent cells of low NK-reactive mice. *Int. J. Cancer*, **28**, 811.

SCHNEIDER, E.L., STANBRIDGE, E.J. & EPSTEIN, C.J. (1974). Incorporation of $^3$H-uridine and $^3$H-uracyl into RNA. A simple technique for the detection of mycoplasma contamination of cultured cells. *Exp. Cell Res.*, **84**, 311.

SCHORLEMMER, H.U., BOSSLET, K. & SEDLACEK, H.H. (1983). Ability of the immunomodulating dipeptide bestatin to activate cytotoxic mononuclear phagocytes. *Cancer Res.*, **43**, 4148.

SCHROIT, A.J. & FIDLER, J.I. (1982). Effects of liposome structure and composition on the activation of the tumoricidal properties of macrophages by liposomes containing muramyl dipeptide. *Cancer Res.*, **42**, 161.

SEBESTENY, A., TAYLOR-PAPADIMITRIOU, J., CERIANI, R., MILLIS, R., SCHMITT, C. & TREVAN, D. (1979). Primary human breast carcinomas transplantable in nude mouse. *J. Natl. Cancer Inst.*, **63**, 1331.

SHARKEY, F.E. & FOGH, J. (1979). Metastases of human tumour in athymic nude mice. *Int. J. Cancer*, **24**, 733.

STANBRIDGE, E. (1984). Use and validity of tumorigenicity assays in immune-deficient animals. In: *Immune-deficient Animals*. (Ed. Sordat), Lausanne: Karger, p. 197.

STILES, C.D., DESMOND, W.D., CHUMAN, L.M., SATO, G. & SAIER, M.H. (1976). Relationship of cell growth behavior in vitro to tumorigenicity in athymic mice. *Cancer Res.*, **35**, 3300.

STUTMAN, O. (1978). Spontaneous, viral and chemically induced tumors in the nude mice. In: *The Nude Mouse in Experimental and Clinical Research*. (Eds. Fogh & Giovanella), New-York: Academic Press, p. 411.

STUTMAN, O., FIGARELLA, E.F., PAIGE, C.J. & LATTIME, E.C. (1980). Natural cytotoxic cells against solid tumours in mice: General characteristics and comparison to natural killer cells. In: *Natural Cell-mediated Immunity against Tumors*. (Ed. Herberman), New-York: Academic Press, p. 187.

THOMASSET, N., QUASH, G. & DORÉ, J.F. (1982). Diamine oxidase activity in human melanoma cell lines with different tumorigenicity in nude mice. *Br. J. Cancer*, **46**, 58.

TVEIT, K.M. & PIHL, A. (1981). Do cell lines in vitro reflect the properties of the tumours of origin: A study of lines derived from human melanoma xenografts. *Br. J. Cancer*, **44**, 775.

UENISHI, N., IDA, N., KAJITA, A. & USUKI, K. (1983). Host-mediated antitumor effect of interferon in nude mice transplanted with human tumor. *Gann*, **74**, 730.

WALKER, M.J., CHANDHURI, P.K., DAS GUPTA, T.K. & BEATTIE, C.W. (1980). Influence of host sex on the growth of human melanoma. *Proc. Soc. Exp. Biol. Med.*, **165**, 96.

WATANABE, S., SHIMOSATO, Y., KAMEJA, T. & 4 others. (1978). Leukemic distribution of a human acute lymphocytic leukemia cell line in nude mice conditioned with whole-body irradiation. *Cancer Res.*, **38**, 3494.

WHITE, L., MEYER, P.R. & BENEDICT, W.F. (1984). Establishment and characterization of a human T-cell leukemia line (LALW-2) in nude mice. *J. Natl Cancer Inst.*, **72**, 1029.

WOLFE, S.A., TRACEY, D.E. & HENNEY, C.S. (1976). Induction of natural killer cells by BCG. *Nature*, **262**, 584.